# The imperative of diversity and equity for the adoption of responsible AI in healthcare

**DOI:** 10.3389/frai.2025.1577529

**Published:** 2025-04-16

**Authors:** Denise E. Hilling, Imane Ihaddouchen, Stefan Buijsman, Reggie Townsend, Diederik Gommers, Michel E. van Genderen

**Affiliations:** ^1^Department of Gastrointestinal Surgery and Surgical Oncology, Erasmus MC Cancer Institute, University Medical Center, Rotterdam, Netherlands; ^2^Erasmus MC Datahub, University Medical Center, Rotterdam, Netherlands; ^3^Department of Adult Intensive Care, Erasmus MC, University Medical Center, Rotterdam, Netherlands; ^4^Faculty of Technology, Policy and Management, Delft University of Technology, Delft, Netherlands; ^5^SAS Worldwide Headquarters, Cary, NC, United States; ^6^National Artificial Intelligence Advisory Committee, Washington, DC, United States

**Keywords:** artificial intelligence, healthcare, bias, diversity, equity

## Abstract

Artificial Intelligence (AI) in healthcare holds transformative potential but faces critical challenges in ethical accountability and systemic inequities. Biases in AI models, such as lower diagnosis rates for Black women or gender stereotyping in Large Language Models, highlight the urgent need to address historical and structural inequalities in data and development processes. Disparities in clinical trials and datasets, often skewed toward high-income, English-speaking regions, amplify these issues. Moreover, the underrepresentation of marginalized groups among AI developers and researchers exacerbates these challenges. To ensure equitable AI, diverse data collection, federated data-sharing frameworks, and bias-correction techniques are essential. Structural initiatives, such as fairness audits, transparent AI model development processes, and early registration of clinical AI models, alongside inclusive global collaborations like TRAIN-Europe and CHAI, can drive responsible AI adoption. Prioritizing diversity in datasets and among developers and researchers, as well as implementing transparent governance will foster AI systems that uphold ethical principles and deliver equitable healthcare outcomes globally.

## 1 Introduction

Despite the potential of Artificial Intelligence (AI) in healthcare, we face the challenge of striking a balance between defining and holding ethical responsibilities and driving innovation. Various studies still reveal significant biases in AI models used in healthcare. For instance, a postpartum depression model resulted in lower diagnosis rates and treatment for Black women (Park et al., 2021), and a recent study found that Large Language Models use pronouns differently across health professions, reinforcing gender stereotypes by predominantly using male pronouns for doctors and surgeons (Menz et al., 2024). These examples underscore the importance of addressing deeply rooted systemic inequities that affect AI’s fairness and outcomes.

## 2 Challenges and solutions in AI equity

To promote diversity and equity in AI, we must not only acknowledge existing systemic injustices but also address data gaps that reflect historical biases in healthcare. For example, randomized controlled trials (RCTs) have predominantly been conducted with men, and often white men, leading to significant gaps in representation for other groups. A 2024 review of 91 clinical text datasets further showed that 73% of the data came from the Americas and Europe, regions that represent only 22% of the global population, with more than half of the datasets being in English (Wu et al., 2024). This lack of global representation highlights a significant bias in the datasets used to train AI systems, which can lead to inequitable outcomes for underrepresented populations. Developers must critically examine the fairness and diversity of the datasets they use. They have a responsibility to identify these gaps and implement solutions, such as augmenting datasets, including underrepresented groups, or applying bias-correction techniques. For example, actively collaborating with institutions in Africa, Asia, and Latin America to integrate local health data can create more robust and representative datasets. In addition to bias in data, AI systems are also shaped by the demographic makeup of their developers. To ensure the responsible development and adoption of AI, it is crucial to acknowledge that the people driving AI innovation in healthcare often do not represent those who are disproportionately burdened by diseases. Most AI researchers today are still white, male, and affiliated with institutions in high-income countries. This lack of diversity reflects broader structural inequities in academic science, which influence both the research questions asked and the solutions proposed. Addressing these inequities requires prioritizing equitable representation both in data and among AI developers and researchers, while building inclusive research cultures that support scientists from marginalized and diverse socio-economic backgrounds to mitigate biases and promote fairer, more equitable outcomes in AI.

**Figure 1:**
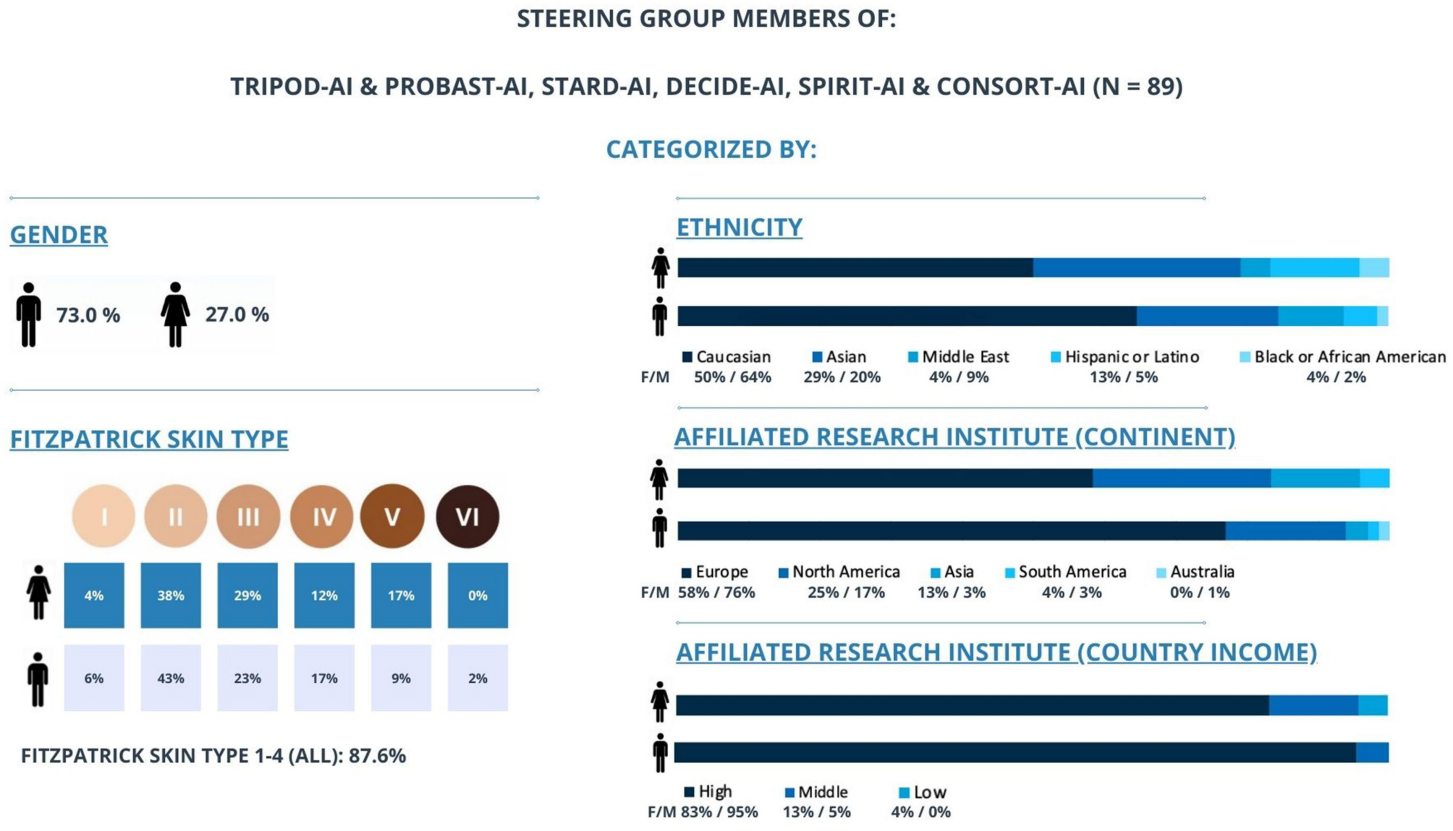
Gender, ethnicity, and geographical distribution of the major medical AI guidelines developers. This figure illustrates how the steering group members of TRIPOD-AI & PROBAST-AI, STARD-AI, DECIDE-AI, SPIRIT-AI & CONSORT-AI are categorized by gender, Fitzpatrick skin type (I–VI), ethnicity, and affiliated research institute (by continent and country income-level) (*n* = 89). The chart shows that a majority of members are male (73.0%) and White (61%), with 72% affiliated with European institutions and 92% based in high-income countries. Fitzpatrick skin types I–IV account for 87.6% of the group, indicating a lack of representation from darker skin types. Collectively, these data highlight potential imbalances in diversity and underscore the importance of broader representation in guideline development.

Ensuring robust evidence, transparency, and accountability throughout the AI model lifecycle is equally critical. Substantial gaps persist in demonstrating the effectiveness of AI prediction algorithms, particularly in evaluating their real-world impact. Initiatives such as the Coalition for Health AI (CHAI) and reporting guidelines play a crucial role in addressing these challenges by fostering broad community consensus and improving accountability (Shah et al., 2024b). Existing guidelines have improved the applicability and reporting of AI in healthcare but continue to fall short in addressing critical ethical considerations. These include algorithmic registration, training and performance requirements, exploration of algorithmic bias, privacy preservation, and AI adoption criteria. Additionally, transparency in the entire lifecycle of AI model development and deployment, including detailed documentation of development decisions, deployment environments, and post-deployment monitoring, should be emphasized to ensure accountability. Factors that, if neglected, risk introducing biases and exacerbating health inequities. An additional limitation of these guidelines is the lack of diversity in the steering groups responsible for developing these AI publishing guidelines, particularly in terms of gender and geographical representation. Demographics analysis show that most members of these steering groups are male, white, and from high-income countries (Figure 1). Improved representation of diverse populations would foster a more inclusive research environment and ensuring equitable, comprehensive research outcomes.

To address these equity challenges effectively, we must prioritize solutions such as federated data access, which enables cross-border data and model-sharing frameworks without the need for physical data transfer (van Genderen et al., 2024). For example, federated learning platforms can allow multiple hospitals across different regions to collaboratively train AI models while keeping patient data local, thereby preserving privacy and enhancing the representativeness of the training data. Secure multi-party computation and robust encryption protocols allow institutions, including those in developing countries, to collaborate in AI model training without compromising data privacy. This approach supports the development of more inclusive and globally diverse health datasets. Additionally, initiatives such as the STANDING Together project encourage systematic collection of demographic variables thus promoting inclusivity and diversity in health datasets (Ganapathi et al., 2022). Moreover, the medical AI community must prioritize the implementation of structural initiatives, such as standardized evaluations, fairness audits and transparent reporting, to ensure that AI systems perform equitably across diverse patient groups. Agreed thresholds for acceptable performance disparities should be established to protect underserved populations from AI-induced harm. Early registration requirements for AI models that influence clinical decisions are also crucial to ensure transparency regarding training data, model performance, and the processes governing model deployment and updates. We therefore applaud networks such as the Trustworthy & Responsible AI Network Europe (TRAIN-Europe) that unifies responsible AI practices in healthcare, not only across Europe but globally, and help organizations to assess and improve their AI maturity. Public-private partnership, with involvement from government bodies like the FDA and NIH, can also promote transparency and accountability, helping fulfill the potential of responsible, equitable AI in healthcare (Shah et al., 2024a).

## 3 Discussion

To achieve the implementation of responsible AI in healthcare, it is not only essential to establish clear standards that evaluate AI system fairness and transparency but also to address structural and institutional factors that contribute to inequities. This includes leveraging diverse health data from different continents and ensuring adequate representation from developing countries to promote diversity, and global equity. Such a unified, multi-institutional, and cross-border effort must prioritize the needs of marginalized communities. By adopting this approach, we can develop AI systems that uphold ethical principles, mitigate bias, and ensure equitable outcomes for all.

## Data Availability

The raw data supporting the conclusions of this article will be made available by the authors, without undue reservation.
